# Supplementary material for: Combined deletions of IHH and NHEJ1 cause chondrodystrophy and embryonic lethality in the Creeper chicken
Source: Commun Biol. 2020 Mar 25;3:144. doi: 10.1038/s42003-020-0870-z (PMC7096424; doi:10.1038/s42003-020-0870-z)
Supplement: Supplementary file 1 — Supplementary Information [file 42003_2020_870_MOESM1_ESM.pdf]

Supplementary Figures

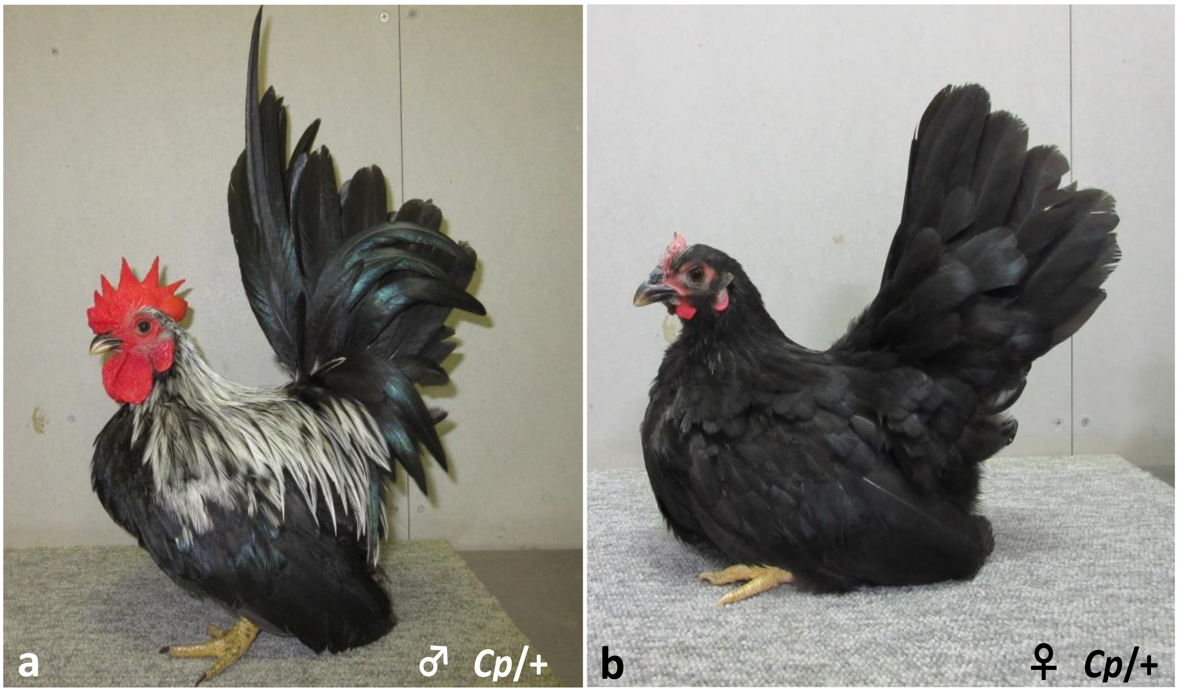

Supplementary Fig. 1 Phenotypes of Creeper chickens. Whole body images of heterozygous (*Cp*/+) male (**a**) and female (**b**) from the Japanese bantam (JB) strain.

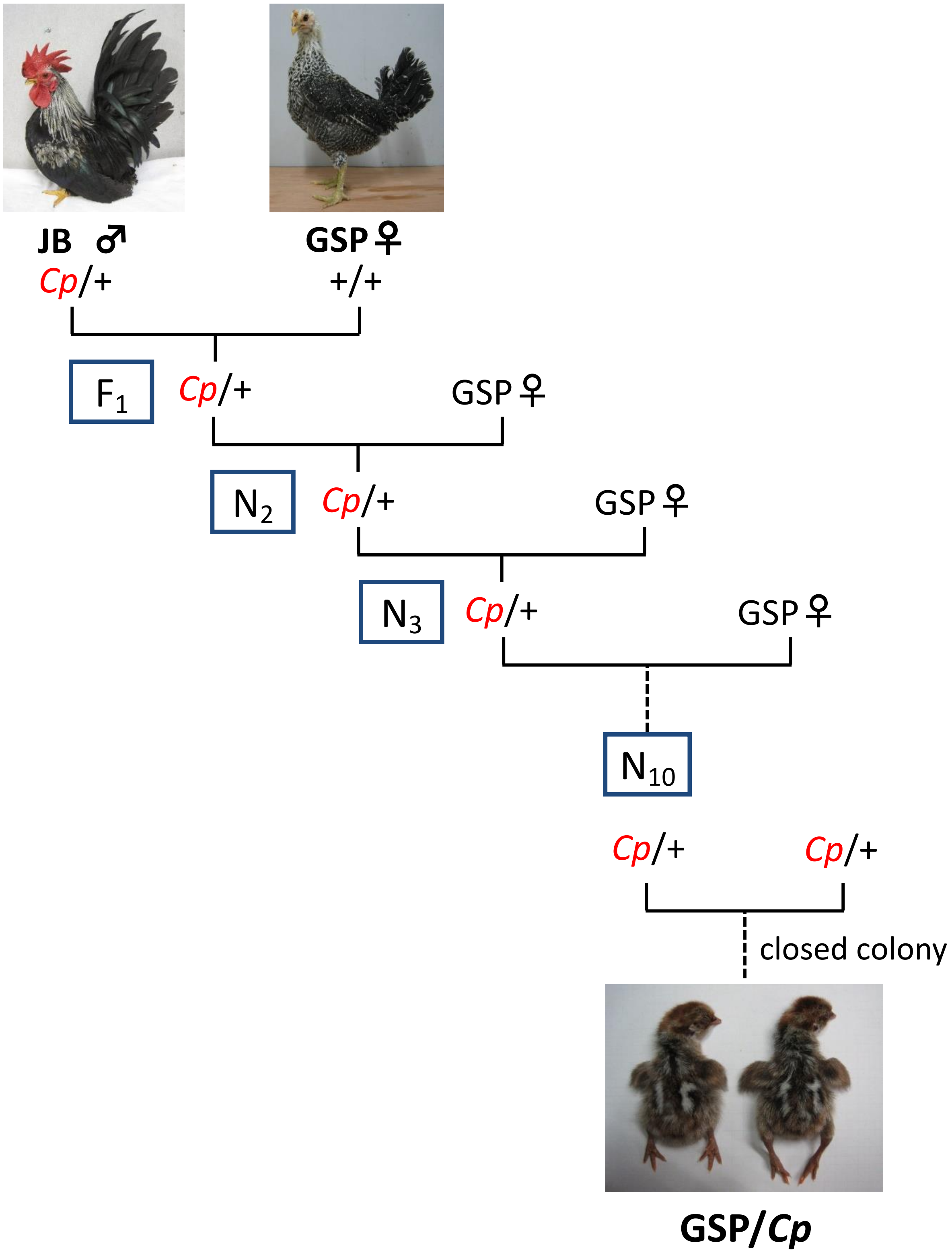

Supplementary Fig. 2 Mating scheme for establishing a congenic strain of the *Cp* gene (GSP/*Cp*). F_1 hybrids were obtained by mating a female of the GSP strain with a male of the JB strain that was heterozygous for the *Cp* allele. The congenic strain of the *Cp* gene in the GSP genetic background was established by backcrossing an F_1 hybrid male to GSP females, followed by backcrossing with GSP females eight times. This strain has been maintained as a closed colony.

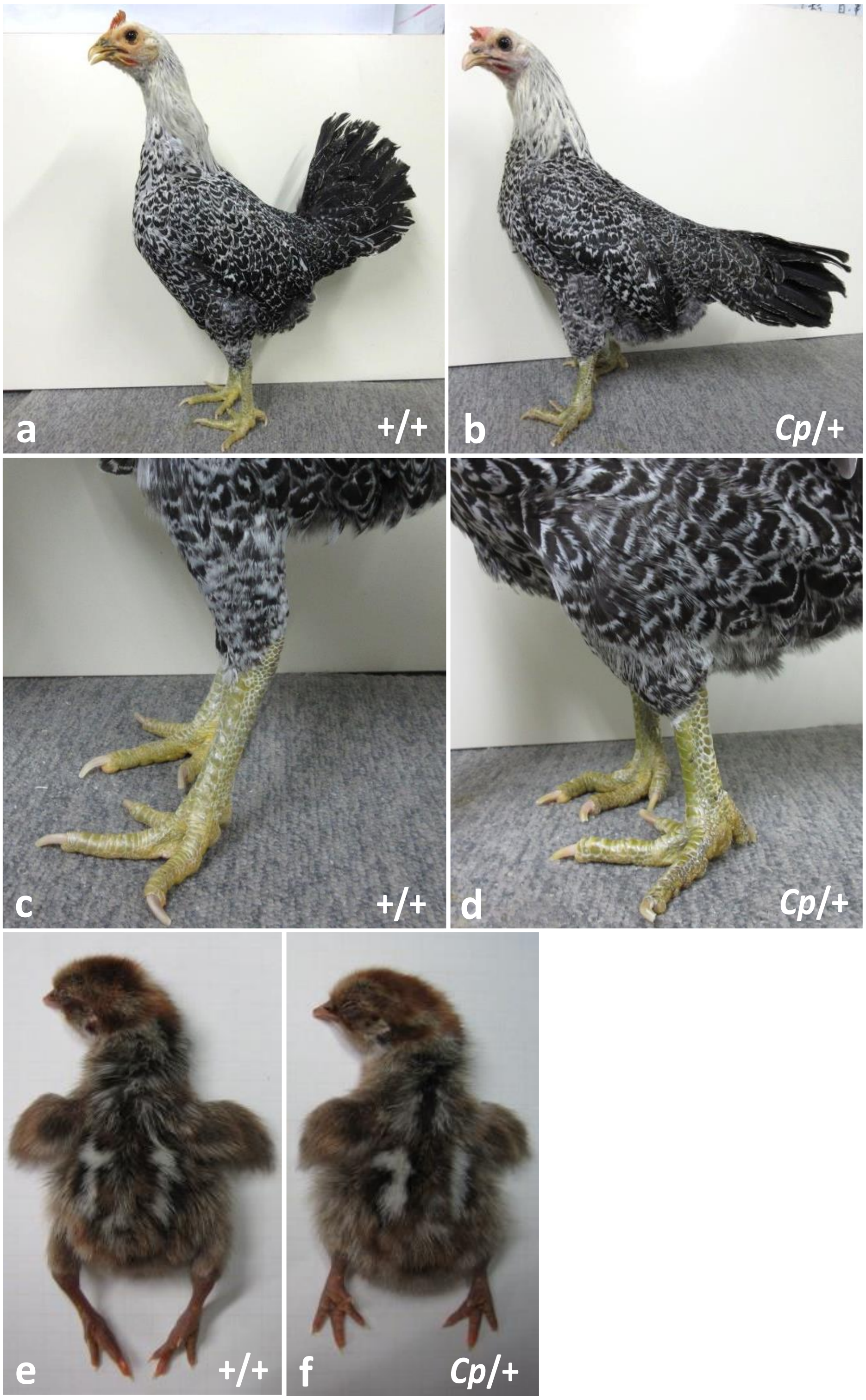

Supplementary Fig. 3 Phenotypes of the wild-type and Creeper chickens in the congenic GSP/*Cp* strain. **a–d** Pictures of whole bodies and legs of a wild-type chicken (+/+) (**a**, **c**) and a Creeper chicken (*Cp*/+) (**b**, **d**). The legs of the Creeper chicken (**d**) are shorter than those of the wild-type chicken (**c**). **e, f** A wild-type chick (+/+) (**e**) and a Creeper chick (*Cp*/+) (**f**) 1 day after hatching.

a

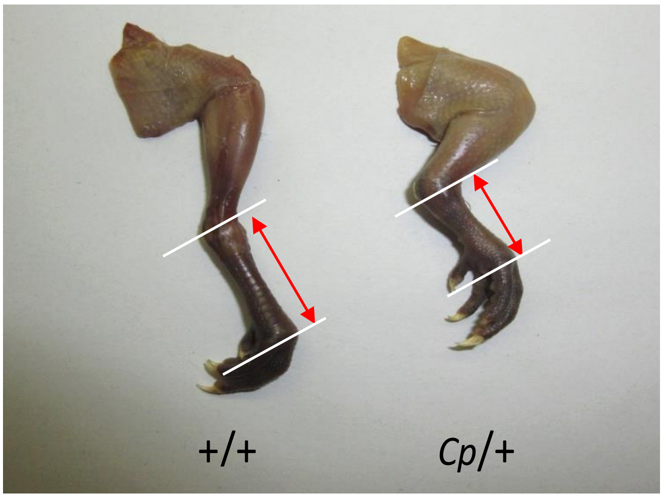

b

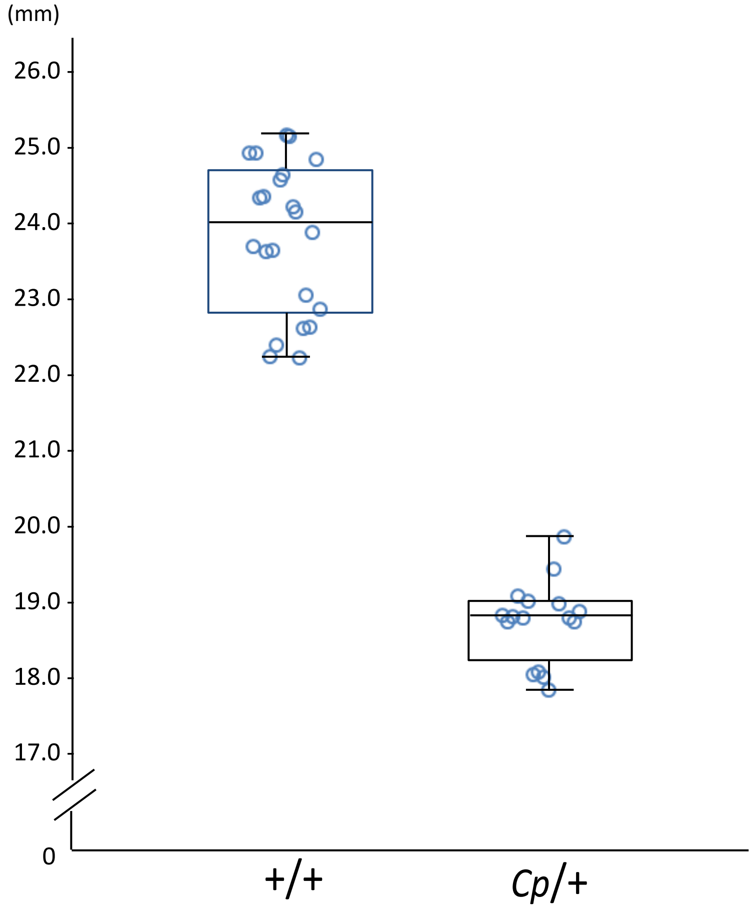

Supplementary Fig. 4 Shank lengths of the wild-type and Creeper chicks in the GSP/*Cp* strain. **a** The right legs of a wild-type chick (+/+) and a Creeper chick (*Cp*/+) 1 day after hatching. The lengths of metatarsal bones (double-headed arrows) were measured using an electronic caliper. **b** Boxes and whisker plots show the lengths of metatarsal bones of wild-type chicks (+/+, n = 22) and Creeper chicks (*Cp*/+, n = 16). Error bars extend to the maximum and minimum values of each group. The shank length of Creeper chicks was approximately 20% shorter (average = 18.8 mm) than that of the wild-type chicks (average = 23.9 mm) (Welch's t-test, $P = 1.55 \times 10^{-20}$).

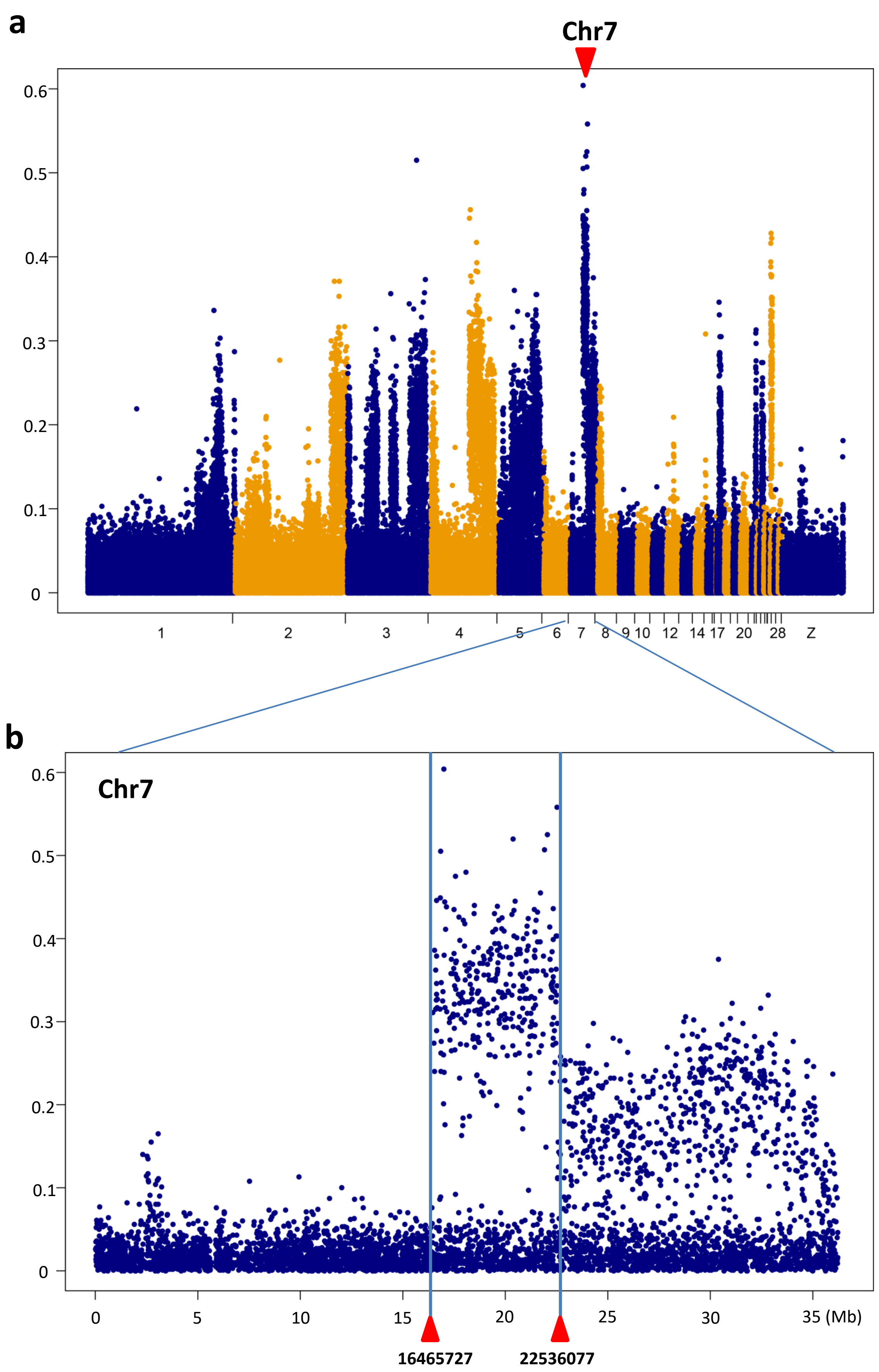

Supplementary Fig. 5 Distribution of genome-wide absRAFdif values obtained using a 600K genome-wide SNP array. **a** The highest peak of absRAFdif values between the wild-type (+/+) and heterozygous (*Cp*/+) chickens located on chromosome 7. **b** The peak of absRAFdif values was localized within 6.1 Mb at nucleotide position 16465727 – 22536077 in the chicken reference genome Gallus_gallus-4.0 (17032895 – 23162069 in Gallus_gallus-5.0).

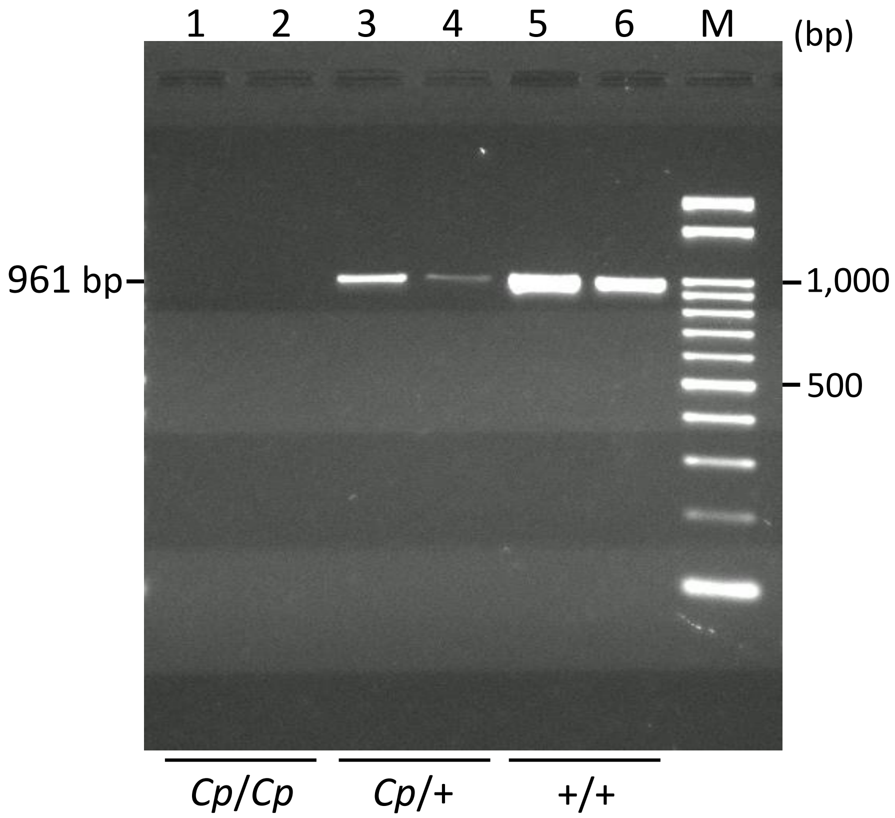

Supplementary Fig. 6 Detection of the genomic deletion of the *IHH* gene at the *Cp* locus. The 961 bp fragment was amplified from the wild-type (+/+) and heterozygous (*Cp*/+) chickens, but not amplified in the *Cp*/*Cp* homozygous embryos using a pair of primers designed in the fifth intron and sixth exon of the *IHH* gene. M, molecular size marker: Gene Ladder 100 (0.1 – 2 kbp) (NIPPON GENE, Tokyo, Japan).

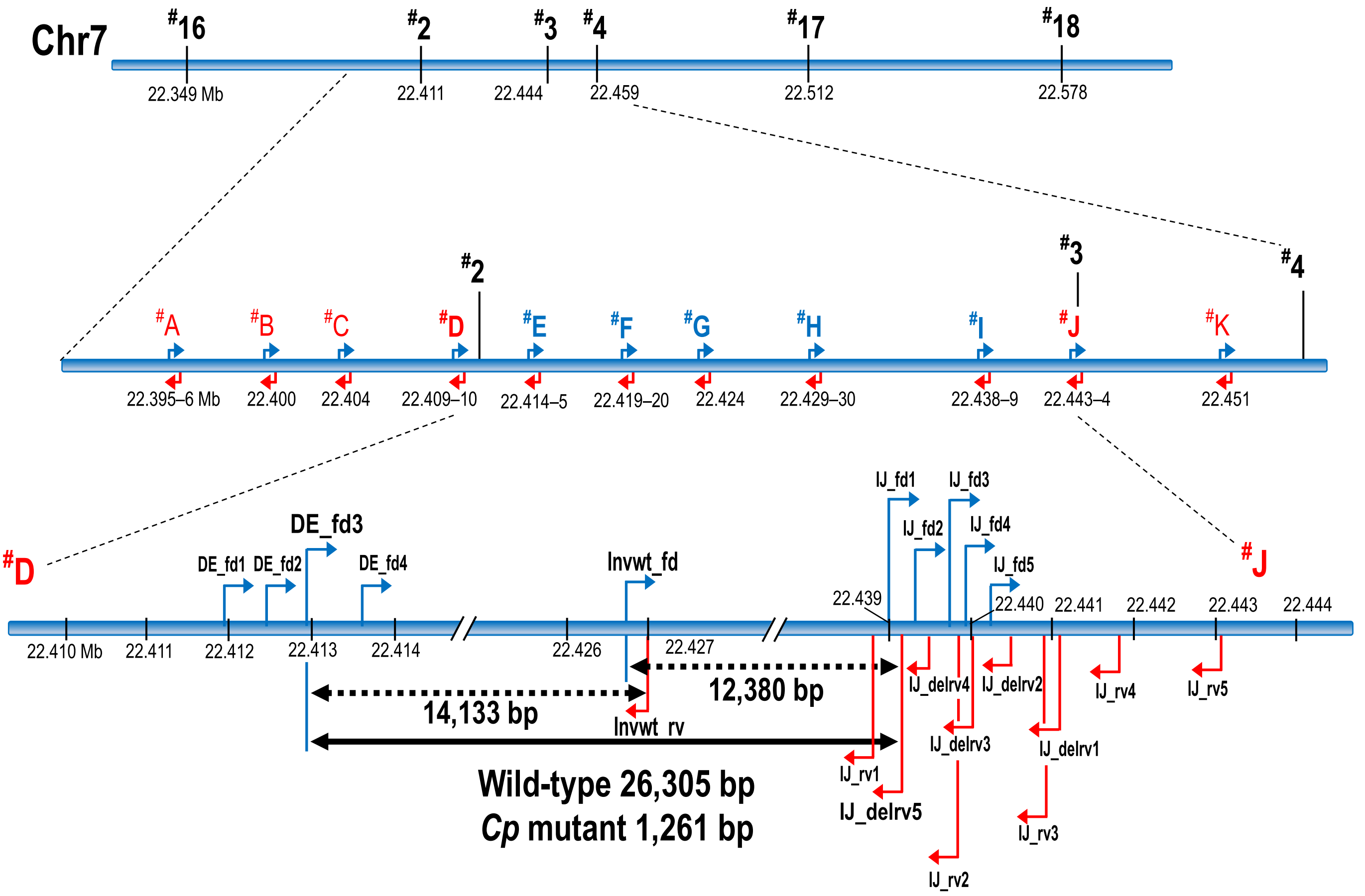

Supplementary Fig. 7 The locations of PCR primers used for detecting deletions at the *Cp* locus. Six SNPs (#2, #3, #4, #16, #17, and #18) in the vicinity of the *Cp* locus were used as anchors for detecting deletions. The key-shaped arrows indicate the positions of PCR primers.

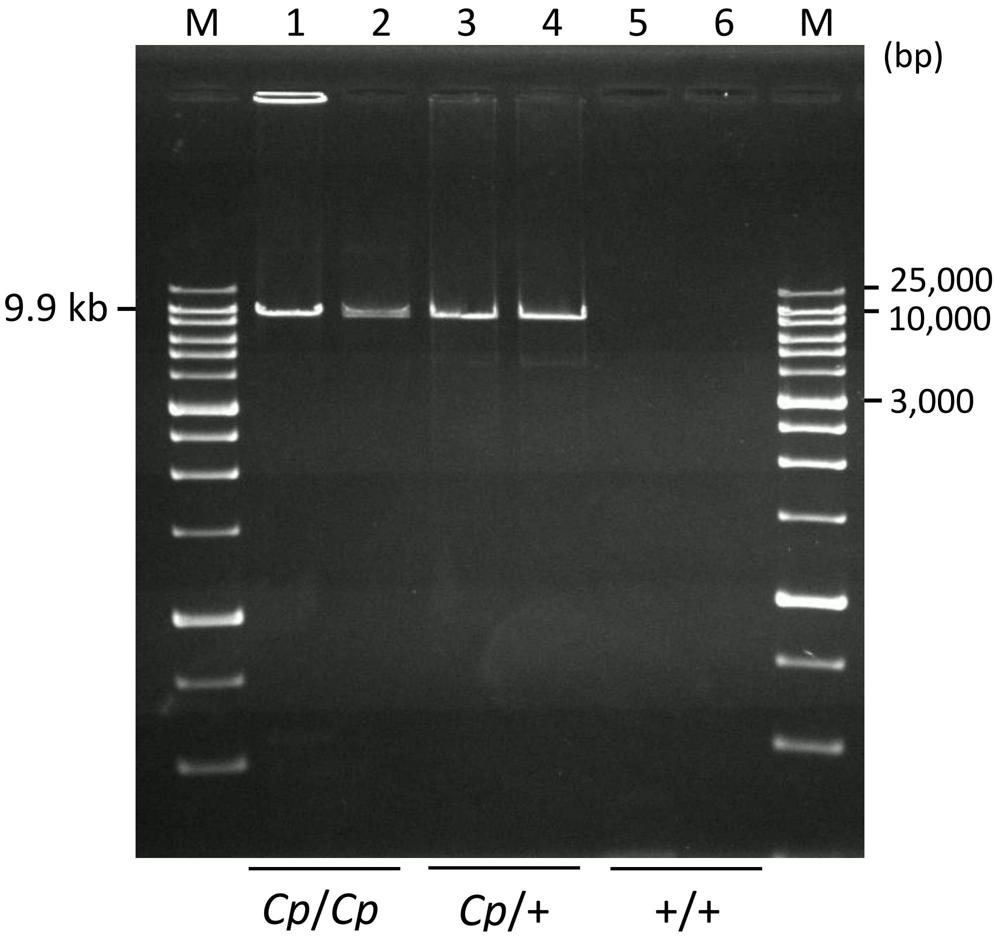

Supplementary Fig. 8 PCR amplification for detecting the deletion at the *Cp* locus. A 9.9 kb PCR product was only amplified from the *Cp*/+ chickens and the *Cp*/*Cp* embryos but not in the wild-type (+/+) chickens by LA-PCR using primers #D_fd and #J_rv in the 35 kb region at 22.409 – 22.444 Mb in Gallus_gallus-5.0. M, molecular size marker: ExcelBand XL 25 kb DNA Ladder, Broad Range (up to 25 kb) (SMOBIO Technology, Hsinchu, Taiwan).

```
→ DE_fd3 (22412905-22412930)
TTCGTTTTCT GGCAACATGC AGCTCCGAGC TGAGCGCATT GGTCAGGCTG   50
TGCGCAGGGC TTGGGCTCCC AGTGCTGTCC CCTGGCTGCT CCCGTGCTCC  100
GAAAAATGTA ATGGTGCTCT TCCAGCATCA CTGCCAGACC AGTTTCATCC  150
CTCTCTGGCT GCGCTTCCAC CTTGCCAAGC AGGGTAAGCT GGCACCACGG  200
GCACACCTGT GCCTGGCTGA AGCTGGTAGC AATTCGGAGA GATCCAGAGG  250
CAGATGTCCT GGTGCCTTGG TGCTCTGTCT TTCTGCTCCT CCAGCCAAAA  300
      22413167       22413168-22426650 (13,483 bp)      22426651
TCTGTCTCCA TCACCCAGCG CGGACCTGCA GCTATTCCAG CCTGGCCCtc  13750
ccgtgtgccc acccgaggga tgtgagggga cgtcccggcc gaggcggtgt  13800
gcgtagggct gtcccgaggt gctgcctcac ctgggaaggc cacagggaac  13850
ttgcaggggt gcaggcagag tgcagccgga ccccttcccc ggcgtgcagc  13900
                          → Invwt_fd (22426831-22426858)
tgggggagac tcaggggggc cccgcgaacc ctcgctcctg aaatcagggt  13950
gttgtattta ctttcccctt ttggcagcta gaaggggaag cccatcccag  14000
cccctcattg ttgtctcagc gagttaatca gatgccataa tcctgttgtg  14050
gaaatatcgg gcctccctgg aaacaatggg gagcgagaat attgtggtta  14100
aatttcaacc agctgcctcc ctgctctgtt tgatccccgc ccgcaggccc  14150
    ← Invwt_rv (22427010-22427037)
gggcccctgg cgagcgggcg gcgcggcggg ggccggagcc ccgggggtgc  14200
cacctcgtgc ccgggggtgt cccgttggcg gggggcgtcg ggccgggcgg  14250
gcggcggggg cggccccgag gggcaggtgg cggggtgggc tgcggggctc  14300
ggccggtggg aggaggctgc gggCGGCCCG GGGCAGGTTC CGCGGGGAGG  14350
                    22427228    22427229-22438789 (11,561 bp)
                                     22438790
GGCGCAGAGT GGTCTGTGCT GAGCCAACAC TCCAGGAATT TAATTTGTTT  25900
AATATATTCC CTGGGAAAGA AGAAAATAAA TAAGTGGCAC CTTGCGTGCC  25950
CCCATGGCTC ACTCTCGCAA CGGAGGTGTC AGCTTAACGA GCCGAAGCCC  26000
TCAGCCTGAT TAACCAGCCG CTGCTCCCGC TAATAGGGAG CCACCGAGCC  26050
GCGTGATTGA TGGAAGGGAG CATTACCCCA GCAGCACGCT GCTGCGAGGA  26100
GGTGCTGTGC CTCAGCCCCC TGCTCCTGCC CAGGCACAGG TCAAGGGCAG  26150
AGACGGCTCC CACCTCCCTG GGCAGATCCC TCTGCCCCAC TGCTCTGCCA  26200
GGCCGGGCTT CGGGACTGCA TGGCTGAGTG CTGGGTCTTG GCCTTGCTCT  26250
TCCCTGAGGC CGCGTGGGGA CACTGAGGCA CAGGGATGTT GACAGGCCAA  26300
GTGCTT                           ← IJ_delrv5 (22439184-22439209)

Wild-type 14,133 bp
Cp mutant unamplified

Wild-type 12,380 bp
Cp mutant unamplified
```

Supplementary Fig. 9 The nucleotide sequence of the 1,261 bp fragment amplified from the *Cp*/*Cp* embryos with primers DE_fd3 and IJ_delrv5. The inverted 578 bp sequence remains in the 25 kb deleted region in the *Cp* allele, which is located between nucleotide positions 22413167 and 22438790.

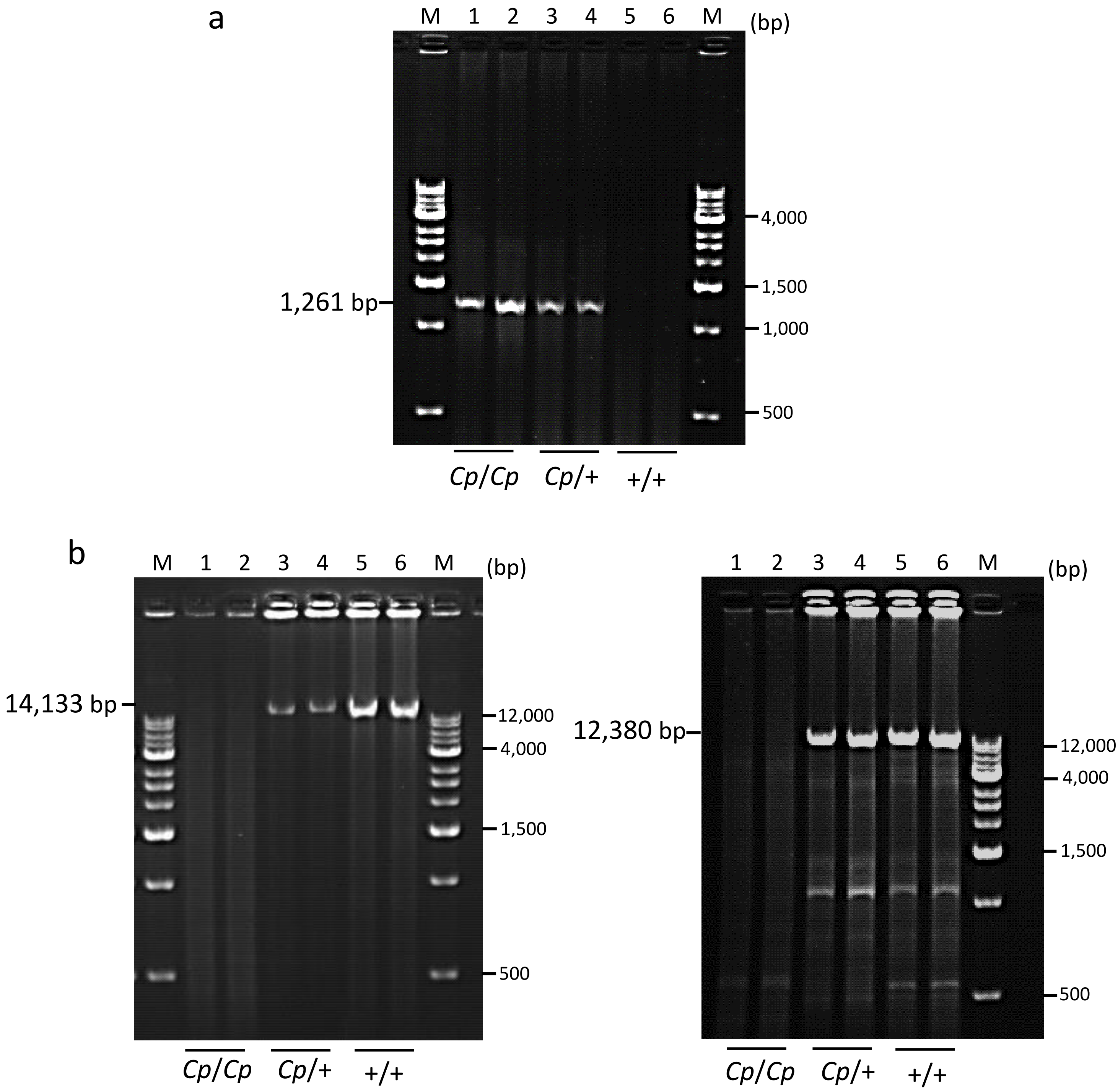

Supplementary Fig. 10 PCR amplification for detecting the deletion at the *Cp* locus. **a** A 1,261 bp PCR product was only amplified from the *Cp*/+ chickens and the *Cp*/*Cp* embryos but not in the wild-type (+/+) chickens by LA-PCR using primers DE_fd3 and IJ_delrv5 in the 26 kb region at 22.413 – 22.439 Mb in Gallus_gallus-5.0. **b** 14,133 bp (left) and 12,380 bp (right) PCR products were only amplified from the wild-type (+/+) and *Cp*/+ chickens but not in the *Cp*/*Cp* embryos by LA-PCR using DE_fd3 and Invwt_rv primer pair and Invwt_fd and IJ_delrv5 primer pair, respectively (see Supplementary Fig. S8 and Supplementary Table 9). M, molecular size marker: DL10001 DNA ladder (500 – 12,000 bp) (Generay Biotech, Shanghai, China).

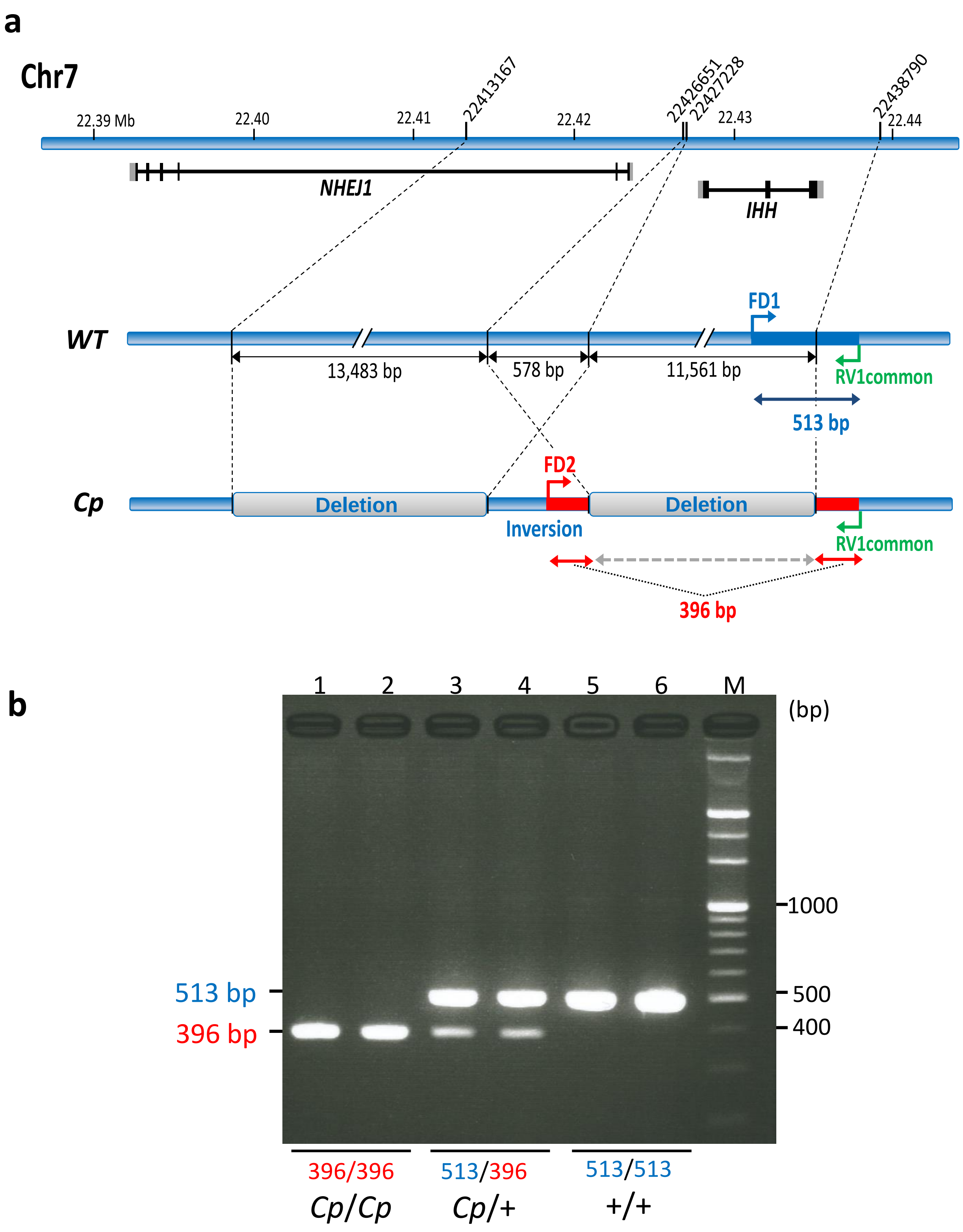

Supplementary Fig. 11 Genotyping of the wild-type and Creeper alleles using a PCR assay. **a** A physical map of two deletions and the remaining inverted fragment in the deleted region at the *Cp* locus. The breakpoints located in the fourth intron of *NHEJ1* and behind the 3' UTR of *IHH*. The key-shaped arrows indicate the positions of PCR primers, FD1, FD2, and RV1common, used for genotyping. **b** Electrophoretic pattern of the DNA fragments amplified from the three genotypes. A 513 bp fragment was amplified from the wild-type allele by primers FD1 and RV1common, and a 396 bp fragment from the *Cp* allele by primers FD2 and RV1common. M, molecular size marker: Gene Ladder 100 (0.1 – 2 kbp) (NIPPON GENE, Tokyo, Japan).

Supplementary Tables

Supplementary Table 1 Segregation of Creeper and wild-type phenotypes in F_1, F_2, and backcross progeny that were obtained from mating between the JB line and the two wild-type strains, PNP/DO and GSP

Mating		No. of progeny	Phenotype of progeny		Expected ratio	χ^2	*P*
Male (Expected genotype) (No. of individuals)	Female (Expected genotype) (No. of individuals)		Creeper	Wild-type			
JB (*Cp* /+) (1)	PNP/DO (+/+) (1)	2	1	1	–	–	–
F_1 (*Cp* /+) (1)	F_1 (+/+) (1)	113	56	57	1 : 1	0.009	$P<0.05$
JB (*Cp* /+) (1)	GSP (+/+) (1)	24	10	14	1 : 1	0.667	$P<0.05$
F_1 (*Cp* /+) (2)	F_1 (*Cp* /+) (4)	175	118	57	2 : 1	0.043	$P<0.05$
F_1 (*Cp* /+) (2)	F_1 (+/+) (2)	120	62	58	1 : 1	0.133	$P<0.05$
F_1 (*Cp* /+) (1)	GSP (+/+) (1)	15	7	8	1 : 1	0.067	$P<0.05$

Supplementary Table 2 Genotyping of the 25 kb deletion (del) at the *Cp* locus in the E2.5 – 3.0 embryos from mating between *Cp* /+ heterozygotes from the GSP/*Cp* congenic strain, and morphological characteristics of the embryos

Stage of developemt	No. of embryos examined	No. of dead embryos	No. of live embryos	Morphology of live embryos					
				Normal			Abnormal		
				+/+	del/+	del/del	+/+	del/+	del/del
E2.5 – 3.0[a]	110	4	106	27	43	3	3	3	27

[a] Collected 3 days after the start of incubation

Supplementary Table 3 Classification of morphological abnormalities in the E2.5 – 3.0 embryos obtained from a mating between *Cp* /+ heterozygotes from the GSP/*Cp* congenic strain

Genotype	No. of abnormal embryos[a]	Morphological abnormality		
		Hypoplasia of brain	Abnormal blood island formation	Heart hypoplasia
+/+	3	3	0	0
del/+	3	1	2	0
del/del	27	26	19	23

[a] Collected 3 days after the start of incubation in the experiment shown in Supplementary Table 2

Supplementary Table 4 Eighteen haplotype regions that showed high absRAFdif values between the wild-type (+/+) chickens from the GSP strain and the heterozygoous (*Cp* /+) chickens from the GSP/*Cp* strain by SNP genotyping assay

Chromosome	Nucleotide position[a]		Median of absRAFdif values	Haplotype block size (Mb)
	5' region	3' region		
7	16465727	22536077	0.335	6.1
26	394415	2705165	0.24	2.3
4	50446006	69851627	0.221	19.4
7	22677582	35976759	0.191	13.3
3	81609740	104480421	0.187	22.9
2	126787922	148326478	0.181	21.5
17	3395054	6096006	0.178	2.7
4	2426384	3874078	0.172	1.4
3	53045	3228287	0.171	3.2
4	70002537	83960114	0.171	14
5	15847877	55356475	0.167	39.5
3	30919389	40051721	0.164	9.1
21	5795548	6763857	0.159	1
1	170335509	177682674	0.157	7.3
3	57002224	64541911	0.149	7.5
23	2762047	5694912	0.147	2.9
8	2310802	5061393	0.141	2.8
1	193871308	194405013	0.128	0.5

[a] Nucleotide position in the reference genome Gallus_gallus-4.0

Supplementary Table 5 Primer sets of 16 SNP markers used for mapping the *Cp* locus on chromosome 7

Marker		Nucleotide sequence of primer (5' to 3')	SNP position (Gallus_gallus-4.0)	SNP position (Gallus_gallus-5.0)	SNP[a] (JB/GSP)	Size of PCR product (bp)	Annealing temparature (°C)	Restriction enzyme (No. of restriction sites)
CHR7_#5	Forward	AAACAAGACAGAGCCAGCTTCC	2523701	2555424	C/T	458	59.5	*Pst* I (1)
	Reverse	TTGGGTCGTGTAGATAGGCAAG						
CHR7_#6	Forward	AGAAAGACATGCAGCCACAGAG	7468194	7977228	C/A	424	59.5	*Eco* RI (1)
	Reverse	TTGGGCTTAGCAAAACAGCACC						
CHR7_#19	Forward	ACCATATTTCTTCAATAAG	15711523	16274591	C/T	317	47.5	*Hin* dIII (1)
	Reverse	AGCTTGTAGACTACGTGAGC						
CHR7_#8	Forward	AGGTAGAGTCTGTAGGTCTCAG	17462758	18049452	C/T	438	59.5	*Pst* I (1)
	Reverse	TGGTCATAATGAGTGGCAGCAG						
CHR7_#9	Forward	AAGTGCTCCCAAGTAGGATCAG	19709516	20305921	A/G	459	59.5	*Pst* I (1)
	Reverse	ACTTACCCTTCCACTCTCCTAC						
CHR7_#14	Forward	TTCAGTGGGATTTCTCAGGAGC	21435690	22049676	T/G	758	59.5	*Pst* I (1)
	Reverse	TCTCTTCAGGAGATTGAGACGG						
CHR7_#15	Forward	TGTCCTCGTAAACGAAGCTCAG	21647789	22268282	A/G	732	58.5	*Nme* AIII (1)
	Reverse	ATGCCCCATGATCATGTGCATG						
CHR7_#16	Forward	TAAAAGCTGGACTGCTGTAGCC	21726140	22348862	T/G	662	59.5	*Nde* I (1)
	Reverse	TTCCTTCAGGAAGCCGATGAAG						
CHR7_#2	Forward	AACTGTGAGCCGATGAAGCAAG	21783101	22411354	A/G[b]	654	59.5	*Bci* VI (1)
	Reverse	AGCACAACCGGCTTCCAATTAG						
CHR7_#3	Forward	AGATTTTCTCCAGCTCTCGTGG	21815955	22444190	C/T[b]	656	59.5	*Pml* I (1)
	Reverse	AAACCTTCTTGCTCCTGCATGC						
CHR7_#4	Forward	ATTTCTCCAGGAGCTGCATGGG	21830848	22458582	C/G	449	59.5	*Pvu* II (2 sites including 1 common site)
	Reverse	TCACAGGAAACTTCGGAGGCAG						
CHR7_#17	Forward	ATGTGGGAGTACCATGTCATGC	21884855	22512418	T/C	782	59.5	*Tsp* 45I (1)
	Reverse	AGCCCCATAGGGAAACTCTTTC						
CHR7_#18	Forward	ACTCAGCACCTTTAGGACTTGC	21951698	22578237	T/C	922	59.5	*Hph* I (2 sites including 1 common site)
	Reverse	AGCCAGCAAGTATGGGATTTCC						
CHR7_#11	Forward	TTCCAAGTGACTCCACCATCTC	23830947	24456802	T/C	486	59.5	*Pst* I (1)
	Reverse	TCCTCTCTTTCAGGGTAGAAGG						
CHR7_#12	Forward	ATCACTGAGAGGCTTCTCGTTG	30424765	31088900	C/G	456	59.5	*Hin* dIII (2 sites including 1 common sit[illegible]
	Reverse	ATTGACCACTCAGAAGGCTCTG						
CHR7_#13	Forward	AGCTGTCCAGGAAAGCCTTATC	35185180	35875738	A/G	424	59.5	*Pst* I (1)
	Reverse	AAGCAGGTTTGCACTTGGTAGG						

[a] SNPs detected between the JB strain and the GSP strain

[b] SNPs detected between the JB strain and the GSP strain in the deleted region

Supplementary Table 6 Linkage analysis of the *Cp* locus using 16 SNP markers and three microsatellite markers on chromosome 7

Marker	Position of marker (Gallus_gallus-4.0)	Position of marker (Gallus_gallus-5.0)	Rough mapping using 95 F_2 progeny		Marker	Position of marker (Gallus_gallus-5.0)	Fine mapping using 175 F_2 progeny	
			Map distance (cM)	LOD score			Map distance (cM)	LOD score
CHR7_#5	2523701	2555424						
			21.6	8.7			–	–
CHR7_#6	7468194	7977228						
			29.6	3.0			–	–
CHR7_#19	15711523	16274591			CHR7_#19	16274591		
			3.8	19.5				
CHR7_#8	17462758	18049452						
			2.1	19.0			8.6	33.8
CHR7_#9	19709516	20305921						
			1.1	21.9				
MCW0133	20859924 – 20860054	21473824–21473954			*MCW0133*	21473824–21473954		
			1.1	22.8			1.2	61.6
CHR7_#14	21435690	22049676			CHR7_#14	22049676		
			0.0	26.6			0.0	70.7
CHR7_#15	21647789	22268282			CHR7_#15	22268282		
			0.5	24.4			0.6	65.4
**CHR7_#16**	**21726140**	**22348862**			**CHR7_#16**	**22348862**		
			0.5	24.1			**0.3**	69.8
**CHR7_#2**	21783101	22411354			**CHR7_#2**	22411354		
			0.0	26.1			**0.0**	69.8
***Cp* phenotype**	–	–			***Cp* phenotype**	–		
			0.0	26.1			**0.3**	66.9
**CHR7_#3**	**21815955**	**22444190**			**CHR7_#3**	**22444190**		
			0.0	26.1			0.0	69.5
CHR7_#4	21830848	22458582			CHR7_#4	22458582		
			0.0	26.1			0.0	69.5
CHR7_#17	21884855	22512418			CHR7_#17	22512418		
			0.5	23.8			0.3	66.6
CHR7_#18	21951698	22578237			CHR7_#18	22578237		
			1.1	21.8			1.4	58.8
MCW183	22208672–22208965	22834668–22834961			*MCW183*	22834668–22834961		
			7.3	16.3				
CHR7_#11	23830947	24456802					13.1	24.4
			6.6	20.7				
ADL180	26581206–26581339	27230408–27230541			*ADL180*	27230408–27230541		
			8.4	20.2			–	–
CHR7_#12	30424765	31088900						
			15.4	15.0			–	–
CHR7_#13	35185180	35875738						

Supplementary Table 7 Primer pairs used for identifying the deletion in a candidate region of the *Cp* locus

Primer name	Nucleotide sequence (5' to 3')	Nucleotide position (Gallus_gallus-4.0)	Nucleotide position (Gallus_gallus-5.0)	Size of PCR product (bp)
#A_fd	TTCTACAGAGCTTAGAGCAGCTGG	21766687–21766710	22394939–22394962	646
#A_rv	ATGTTCTGCAGAAACGTCTCCTCC	21767332–21767309	22395584–22395561	
#B_fd	TTAATGAGCGCAAGGTAGCTGTGG	21771370–21771393	22399622–22399645	686
#B_rv	AGTCTGGTTTTAGCACTGTGCAGC	21772055–21772032	22400307–22400284	
#C_fd	TCTTCGCTGTGGACGTTTCCTTTC	21775297–21775320	22403549–22403572	709
#C_rv	TTTAGCCGTGTGTACCGTACAGTC	21776005–21775982	22404257–22404234	
#D_fd	AAACCCAGAAGGGTTTGCAGTGTG	21780960–21780983	22409213–22409236	685
#D_rv	AAACATGCACCAGAAAGAGGCAGG	21781644–21781621	22409897–22409874	
#E_fd	ATTCAGCAACCTGCTCCTGATTGG	21785847–21785870	22414100–22414123	632
#E_rv	ACATGTCTCTTCAGTGGATGCCAG	21786478–21786455	22414731–22414708	
#F_fd	ATCACCTCTATCACCTCTCTCAGC	21790980–21791003	22419233–22419256	756
#F_rv	TAACTGGAAAACAGAGCGAACGGG	21791735–21791712	22419988–22419965	
#G_fd	AAGGGCTCTTCAGCTGAAAGGTTG	21795295–21795318	22423548–22423571	779
#G_rv	TTGAGAGAAAGCAGTGAGCCTGTG	21796073–21796050	22424326–22424303	
#H_fd	AAGCCATTGACCCAACTCCTCATC	21801452–21801475	22429440–22429463	633
#H_rv	AGTCAGACGTTTTGGGAAGGACTG	21802084–21802061	22430072–22430049	
#I_fd	TGAGGAAGGCTTTAATCAGGACCC	21809669–21809692	22437793–22437816	797
#I_rv	TCTTCAAAACCCAATGCCCTGGAG	21810465–21810442	22438589–22438566	
#J_fd	ATTGTTGGGTGAAGCACCTTCACC	21815259–21815282	22443494–22443517	674
#J_rv	TCATGGTTCAGTCCTGGAATCTCC	21815932–21815909	22444167–22444144	
#K_fd	TTAATGCAAGGCAACAGCTGTGGG	21822337–21822360	22450571–22450594	935
#K_rv	AAACAGTGATGAAGAACCGCGTGG	21823271–21823248	22451505–22451482	

Supplementary Table 8 Primers used for amplifying nucleotide sequences in the deletion region of the *Cp* locus

Primer name	Nucleotide sequence (5' to 3')	Nucleotide position (Gallus_gallus-4.0)	Nucleotide position (Gallus_gallus-5.0)
**5’ Breakpoint**			
DE_fd1	TTTGCACAGTACACATCTCTGGGC	21783611–21783634	22411864–22411887
DE_fd2	ATATATCGGCAGGGTTTGTGTCCC	21784167–21784190	22412420–22412443
DE_fd3	TTCGTTTTCTGGCAACATGCAGCTCC	21784652–21784677	22412905–22412930
DE_fd4	TGTGGGCAATGAGGTTTGGTTTCC	21785304–21785327	22413557–22413580
**578 bp region that remains in the *Cp* allele**			
Invwt_fd	AACCCTCGCTCCTGAAATCAGGGTGTTG	21798578-21798605	22426831–22426858
Invwt_rv	TCAAACAGAGCAGGGAGGCAGCTGGTTG	Not applicable	22427037–22427010
**3’ Breakpoint**			
IJ_fd1	ATTGATGGAAGGGAGCATTACCCC	21810835–21810858	22438959–22438982
IJ_fd2	TCGTTGCAGAGACATCAGCAGTC	21811217–21811239	22439341–22439363
IJ_fd3	TTCCTGAATGATGCCAGGCTGTTG	21811582–21811605	22439706–22439729
IJ_fd4	TTGTTTACCTTCCCCATTGGCCTC	21811806–21811829	22439930–22439953
IJ_fd5	TGAACTGCACTCCTAGATGCTACC	21812167–21812190	22440291–22440314
IJ_rv1	AAATTCCTGGAGTGTTGGCTCAGC	21810670–21810647	22438794–22438771
IJ_delrv5	AAGCACTTGGCCTGTCAACATCCCTG	21811085–21811060	22439209–22439184
IJ_delrv4	ACTGGATAAATCAGCTGCTCCTCC	21811397–21811374	22439521–22439498
IJ_rv2	ATAGTGCTTAATCAGCCCTGAGGC	21811720–21811697	22439844–22439821
IJ_delrv3	TTTGCACCCAACCTACCGTTTTGG	21811973–21811950	22440097–22440074
IJ_delrv2	AAGAGCAAACATCCACGTGGACAC	21812334–21812311	22440458–22440435
IJ_rv3	ATCAGAGCTGGATTAAGGTGTCCG	21812731–21812708	22440855–22440832
IJ_delrv1	ATGGGACAACTTCCTCCTTCTG	21812939–21812918	22441063–22441042
IJ_rv4	TTCCTCTTTGCACCTCTTTGGCAC	21813656–21813633	22441780–22441757
IJ_rv5	AACGGCTCCCACGTTTGTAAGAAC	21814776–21814753	22443127–22443104

Supplementary Table 9 Genotype-phenotype association between the 25 kb deletion (del) and the Creeper phenotype in F_2 progeny of a cross between the JB and GSP strains and 22 chicken strains and/or populations from 17 breeds

Breed (line/population)	Phenotype	No. of samples	Genotype		
			del/del	del/+	+/+
Japanese bantam (JB) (embryo)[a]	embryonic lethal[b]	7	7		
Japanese bantam (JB) (chick)	Creeper	20		20	
	wild-type	18			18
F_2 (*Cp* /+ × *Cp* /+)[c]	Creeper	118		118	
	wild-type	57			57
F_2 (*Cp* /+ × +/+)[c]	Creeper	62		62	
	wild-type	58			58
Miyaji-dori	Creeper	2		2	
	wild-type	1			1
Jitokko	Creeper	6		6	
	wild-type	10			10
Ehime-jidori (EJ)	wild-type	8			8
Cochin bantam (CB)	wild-type	2			2
Shokoku	wild-type	3			3
Polish bantam	wild-type	4			4
Fayoumi (GSP)	wild-type	15			15
Fayoumi (PNP/DO)	wild-type	15			15
Fayoumi (GSN/1)	wild-type	8			8
Fayoumi (YL)	wild-type	8			8
Black Minorka (BM-C)	wild-type	4			4
Brown Leghorn (BL-E)	wild-type	4			4
Rhode Island Red (RIR-Y8/NU)	wild-type	4			4
New Hampshire (413)	wild-type	4			4
Dandarawi (DD)	wild-type	4			4
Japanese Silkie (SIL)	wild-type	8			8
Albino (CAL)	wild-type	4			4
White Leghorn (WL-G)	wild-type	4			4
White Leghorn (M/O)	wild-type	4			4
White Leghorn (OS)	wild-type	4			4
Red junglefowl (RJF/NU)	wild-type	3			3

[a] Embryos were collected 12 – 15 days after the start of incubation

[b] All embryos were dead in the shell

[c] Genotyping was conducted for only hatched F_2 progeny obtained from mating between JB males and GSP fema
