# Supplementary material for: Combined deletions of IHH and NHEJ1 cause chondrodystrophy and embryonic lethality in the Creeper chicken
Source: Commun Biol. 2020 Mar 25;3:144. doi: 10.1038/s42003-020-0870-z (PMC7096424; doi:10.1038/s42003-020-0870-z)
Supplement: Supplementary file 2 — Descriptions of Additional Supplementary Files [file 42003_2020_870_MOESM2_ESM.pdf]

**Supplementary Data** 1. Genotypes of 119 probes from the SNP array in a candidate genomic region of the Creeper trait in four chickens and/or embryos with three different genotypes
