# Supplementary material for: Combined deletions of IHH and NHEJ1 cause chondrodystrophy and embryonic lethality in the Creeper chicken
Source: Commun Biol. 2020 Mar 25;3:144. doi: 10.1038/s42003-020-0870-z (PMC7096424; doi:10.1038/s42003-020-0870-z)
Supplement: Supplementary file 4 — Reporting Summary [file 42003_2020_870_MOESM4_ESM.pdf]

nature research

Corresponding author(s): Yoichi Matsuda
Last updated by author(s): Feb 10, 2020

Reporting Summary

Nature Research wishes to improve the reproducibility of the work that we publish. This form provides structure for consistency and transparency in reporting. For further information on Nature Research policies, see Authors & Referees and the Editorial Policy Checklist.

Statistics

For all statistical analyses, confirm that the following items are present in the figure legend, table legend, main text, or Methods section.

n/a	Confirmed	
☐	☒	The exact sample size (n) for each experimental group/condition, given as a discrete number and unit of measurement
☐	☒	A statement on whether measurements were taken from distinct samples or whether the same sample was measured repeatedly
☐	☒	The statistical test(s) used AND whether they are one- or two-sided<br>*Only common tests should be described solely by name; describe more complex techniques in the Methods section.*
☒	☐	A description of all covariates tested
☒	☐	A description of any assumptions or corrections, such as tests of normality and adjustment for multiple comparisons
☐	☒	A full description of the statistical parameters including central tendency (e.g. means) or other basic estimates (e.g. regression coefficient) AND variation (e.g. standard deviation) or associated estimates of uncertainty (e.g. confidence intervals)
☐	☒	For null hypothesis testing, the test statistic (e.g. F, t, r) with confidence intervals, effect sizes, degrees of freedom and P value noted<br>*Give P values as exact values whenever suitable.*
☒	☐	For Bayesian analysis, information on the choice of priors and Markov chain Monte Carlo settings
☐	☒	For hierarchical and complex designs, identification of the appropriate level for tests and full reporting of outcomes
☒	☐	Estimates of effect sizes (e.g. Cohen's d, Pearson's r), indicating how they were calculated

Our web collection on statistics for biologists contains articles on many of the points above.

Software and code

Policy information about availability of computer code

Data collection	linkage mapping: Axiom® Genome-Wide Chicken Genotyping Array (Affymetrix)
Data analysis	linkage mapping: Genotyping Console version 4.2.0.26 (Affymetrix)<br>sequence mapping: the chicken reference genome Gallus_gallus-4.0/galGAl4 and Gallus_gallus-5.0/galGAl5 (http://genome.ucsc.edu/cgi-bin/hgBlatI)

For manuscripts utilizing custom algorithms or software that are central to the research but not yet described in published literature, software must be made available to editors/reviewers. We strongly encourage code deposition in a community repository (e.g. GitHub). See the Nature Research guidelines for submitting code & software for further information.

Data

Policy information about availability of data

All manuscripts must include a data availability statement. This statement should provide the following information, where applicable:

- Accession codes, unique identifiers, or web links for publicly available datasets
- A list of figures that have associated raw data
- A description of any restrictions on data availability

Data is available from the corresponding author upon reasonable request.

Field-specific reporting

Please select the one below that is the best fit for your research. If you are not sure, read the appropriate sections before making your selection.

☒ Life sciences ☐ Behavioural & social sciences ☐ Ecological, evolutionary & environmental sciences

For a reference copy of the document with all sections, see nature.com/documents/nr-reporting-summary-flat.pdf

Life sciences study design

All studies must disclose on these points even when the disclosure is negative.

Sample size	No statistics to determine sample size were used. However, sample size is sufficient for genetic linkage mapping and genotype-phenotype association.
Data exclusions	All data were included.
Replication	Essential PCR and nucleotide sequencing were independently replicated by two lab members.
Randomization	No statistical randomization methods were used because there were no data that were required for randomization in this study.
Blinding	No blinding was used for data collection. However, we used double-blind test for replicating key results.

Reporting for specific materials, systems and methods

We require information from authors about some types of materials, experimental systems and methods used in many studies. Here, indicate whether each material, system or method listed is relevant to your study. If you are not sure if a list item applies to your research, read the appropriate section before selecting a response.

Materials & experimental systems

n/a	Involved in the study	
☐	☒	Antibodies
☐	☒	Eukaryotic cell lines
☒	☐	Palaeontology
☐	☒	Animals and other organisms
☒	☐	Human research participants
☒	☐	Clinical data

Methods

n/a	Involved in the study	
☒	☐	ChIP-seq
☒	☐	Flow cytometry
☒	☐	MRI-based neuroimaging

Antibodies

Antibodies used	mouse anti-XLF monoclonal antibody (clone name: D-1, cat#sc-166488, Santa Cruz Biotechnology)<br>rabbit anti-GFP polyclonal antibody (FL, cat#sc-8334, Santa Cruz Biotechnology)<br>mouse β-actin monoclonal antibody (AC-15, cat#A5441, Sigma-Aldrich)<br>mouse anti-γH2AX monoclonal antibody (JBW301, cat#05-636, Sigma-Aldrich)<br>In Situ Cell Death Detection Kit, Fluorescein (cat#11684795910, Roche Diagnostics)<br>Anti-Digoxgenin-AP, Fab fragments from sheep (cat#11093274910, Sigma-Aldrich)
Validation	mouse anti-XLF monoclonal antibody: PMID 30250067 Craxton, A. et al. Nat Commun 9: 3877, 2018.<br>rabbit anti-GFP polyclonal antibody: PMID 27126587 Petsalaki, E. et al. Nat Commun 7: 11451, 2016.<br>mouse β-actin monoclonal antibody: PMID 15048076. Radhakrishnan, S.K. et al. Oncogene 23: 4173–4176, 2004.<br>mouse anti-γH2AX monoclonal antibody: PMID 27518625 Nishihara K et al. Methods Mol Biol 1473: 71–76, 2016.<br>In Situ Cell Death Detection Kit, Fluorescein: validation of TUNEL staining was performed using control section of the chick limb bud which shows apoptotic cells at the distal side in this study.<br>Anti-Digoxgenin-AP, Fab fragments from sheep: validation of alkaline phosphatase reaction of in situ hybridization was perfumed using sense RNA probe which does not show any specific staining in this study.

Eukaryotic cell lines

Policy information about cell lines

Cell line source(s)	Three primary cultured cell lines from the wild-type E3 chicken embryos, which transiently expressed an EYFP-tagged chicken NHEJ1 chimeric protein (EYFP-NHEJ1), EYFP-tagged chicken NHEJ1 mutant chimeric protein (EYFP-NHEJ1mt), or control protein (EYFP), were originally established in our laboratory.

Authentication | All three cell lines were authenticated in this study.

Mycoplasma contamination | The cell lines were not tested for mycoplasma contamination.

Commonly misidentified lines (See ICLAC register) | N/A

Animals and other organisms

Policy information about studies involving animals; ARRIVE guidelines recommended for reporting animal research

Laboratory animals	Three chicken strains (JB, GSP, and PNP/DO) and a congenic strain of the Cp gene (GSP/Cp) in the GSP background were supplied from the National BioResource Project Chicken/Quail, Nagoya University, Japan. F1, F2, and/or backcross generations were obtained by mating of the JB strain with two wild-type strains (PNP/
Wild animals	This study did not involve wild animals.
Field-collected samples	N/A
Ethics oversight	Animal care and all experimental procedures were approved by the Animal Experiment Committee, Graduate School of Bioagricultural Sciences, Nagoya University (approval no. 2014021406). Experiments were conducted according to Regulations on Animal Experiments at Nagoya University.

Note that full information on the approval of the study protocol must also be provided in the manuscript.
